# Supplementary material for: Technical Aspects of Coenzyme Q10 Analysis: Validation of a New HPLC-ED Method
Source: Antioxidants (Basel). 2022 Mar 10;11(3):528. doi: 10.3390/antiox11030528 (PMC8944485; doi:10.3390/antiox11030528)
Supplement: Supplementary file 1 [file antioxidants-11-00528-s001.zip › Supplementary Material 1.pdf]

**Explanation Reports**
**-Annual Report-**

# **Annual Report :** lay-out and underlying statistics

**Explanation of the numbers**

This report is made once a year and based on all data submitted during the annual cycle. Outlyers (results exceeding the mean +/- 7SD) are removed before the calculations. The first column lists all analytes in the scheme. Column 2 deals with accuracy: you can see your mean outcome in that year in comparison to the mean of all labs.
The third column deals with precision. From the four hidden duplicates the CV is calculated according to:

$$CV = \frac{\sqrt{\frac{\sum(\Delta)^2}{n}}}{\bar{x}\sqrt{2}} \times 100\%$$

CV = Coefficient of Variation
$\Delta$ = Difference in the duplicate
*n* = number of duplicates
$\bar{x}$ = mean of results
Again the lab's precision is compared with the median CV of other labs (precison of your lab and of all labs, respectively).
Column 4 deals with the linearity. For each laboratory the coefficient of regression r is calculated with the weighed amounts on the x-axis and the submitted results on the y-axis. The lab's r and the median r of all labs are listed.
Column 5 deals with the recovery. Linearity calculation in column 4 also revealed the relation y = ax + b between submitted results (y) and weighed amounts (x). The slope a is multiplied with 100% and is the recovery of weighed amounts. Again recovery of the lab and the median recovery of all labs is encountered.
The last column shows general data of all labs: the number of labs who submitted results (n) and the Interlab CV calculated according to

$$InterlabCV = \frac{\sqrt{\frac{\sum SD^2}{n}}}{\bar{x}}$$

CV = Interlab CV in the respective specimens of the cycle
SD = standard deviation
*n* = number of specimens in a cycle
*x* = mean of results

The Interlab CV is an indication of the state of the art of harmonisation of results between labs and as such an indication how urgent efforts to achieve better standardisation are needed!

## Flags Analytical Parameters

In the annual report (below) the four analytical parameters accuracy – precision – linearity – recovery are shown. Parameters that can not be calculated are marked with a flag:

- No results submitted: empty white box (example: Malic acid)
- Few Results (defined as <6 out of 8 results): **FR** in a white box (Mevalonic acid)
- Outlying Results (defined as 2 or more outliers): **OR** in a purple box (Glycolic acid)
- Outlying Results & Few Results (defined as <6 results from which at least one an outlier): **ORFR** in a yellow box (Ethylmalonic acid)
- Missing Pair (defined as the situation in which both results of a samplepair are missing or an outlier): **MP** in an amber box (Adipic acid)
- A red flags marks a result in the category “5% worst performances of all labs”. Numeric result in a red box (Suberic acid).

**ERNDIMQA - ANNUAL REPORT**

**Quant.org.acids** [redacted]

[redacted]

**Methodset :** GC-MS

| | Accuracy (mean) | | Precision (CV% duplicates) | | Linearity (r) | | Recovery (%added analyte) | | Data All Labs | |
|---|---|---|---|---|---|---|---|---|---|---|
| Analyte | Your Lab | All Labs | Your Lab | All Labs | Your Lab | All Labs | Your Lab | All Labs | n | Interlab CV |
| 2-OH Glutaric acid | MP | 152 | 32.0% | 20.8% | 0.877 | 0.980 | 89% | 93% | 77 | 35.6% |
| 3 methylglutaric acid | MP | 70.4 | 26.3% | 15.0% | 0.914 | 0.985 | 117% | 101% | 77 | 23.6% |
| 3-OH-3 methylglutaric acid | FR | 66.9 | FR | 24.7% | FR | 0.983 | FR | 77% | 65 | 62.4% |
| 3-OH-Isobutyric acid | OR | 136 | OR | 20.4% | OR | 0.979 | OR | 71% | 53 | 37.0% |
| 3-OH-Isovaleric acid | FR | 78.1 | FR | 39.3% | FR | 0.940 | FR | 112% | 73 | 55.5% |
| 4-OH-Butyric acid | FR | 72.8 | FR | 31.2% | FR | 0.962 | FR | 70% | 63 | 56.7% |
| Adipic acid | MP | 178 | 31.6% | 12.6% | 0.950 | 0.993 | 85% | 101% | 85 | 26.4% |
| Creatinine | | 3258 | | 3.4% | | 0.000 | | 0% | 67 | 5.25% |
| D,L- Glyceric acid | FR | 278 | FR | 22.0% | FR | 0.985 | FR | 83% | 64 | 47.0% |
| Ethylmalonic acid | ORFR | 50.0 | ORFR | 17.1% | ORFR | 0.992 | ORFR | 99% | 89 | 36.3% |
| Fumaric acid | MP | 60.0 | 19.5% | 14.5% | 0.991 | 0.991 | 75% | 100% | 78 | 28.5% |
| Glutaric acid | MP | 112 | 44.0% | 13.1% | 0.878 | 0.994 | 86% | 101% | 90 | 21.8% |
| Glycolic acid | OR | 236 | OR | 20.6% | OR | 0.963 | OR | 89% | 77 | 38.0% |
| Hexanoylglycine | MP | 17.0 | 29.3% | 29.9% | 0.949 | 0.950 | 111% | 100% | 64 | 37.2% |
| Keto-glutaric acid | | 198 | | 21.4% | | 0.992 | | 103% | 73 | 40.1% |
| Malic acid | | 117 | | 19.8% | | 0.995 | | 81% | 53 | 47.4% |
| Methylmalonic acid | 305 | 330 | 31.2% | 11.4% | 0.995 | 0.996 | 95% | 102% | 104 | 27.6% |
| Mevalonic acid | FR | 132 | FR | 21.7% | FR | 0.992 | FR | 82% | 56 | 46.1% |
| Pyroglutamic acid | 353 | 322 | 61.6% | 23.0% | 0.879 | 0.980 | 106% | 85% | 73 | 44.2% |
| Sebacic acid | MP | 55.4 | 15.2% | 19.4% | 0.987 | 0.987 | 91% | 101% | 83 | 35.9% |
| Suberic acid | 123 | 172 | 11.5% | 16.3% | 0.992 | 0.990 | 64% | 105% | 84 | 32.4% |
| Tiglylglycine | MP | 83.6 | 17.0% | 24.9% | 0.975 | 0.987 | 54% | 85% | 57 | 48.0% |
| Vanillactic acid | | 38.5 | | 19.4% | | 0.975 | | 98% | 38 | 33.0% |
| Overall | 260 | 270 | 29.0% | 20.1% | 0.944 | 0.940 | 88% | 89% | 71 | 37.7% |

## Flags Analytes

Acceptable performance of an analyte is marked with a green flag (analyte name in green box) and applies when at least 3 of the 4 analytical parameters show satisfying results:

- 4 satisfying parameters (no flags = approved): Methylmalonic acid
- 3 satisfying parameters (1 flag = approved): Tiglylglycine
- 2 satisfying parameters (2 flags = not approved): Suberic acid (2 red flags) and Glutaric acid (1 red and 1 amber flag).
- 1 satisfying parameter (3 flags = not approved): no example
- 0 satisfying parameters (4 flags = not approved): Glycolic acid

**Poor Performance**
The poor performance policy of ERNDIM is based on the number of flags for the analytical parameters in the annual report. The scientific advisor of the scheme sends a performance support letter to the laboratories with the highest number of flags.

**Annual Report**
A comment to the annual report can be downloaded from the bottom of the report.
