# Supplementary material for: Technical Aspects of Coenzyme Q10 Analysis: Validation of a New HPLC-ED Method
Source: Antioxidants (Basel). 2022 Mar 10;11(3):528. doi: 10.3390/antiox11030528 (PMC8944485; doi:10.3390/antiox11030528)

ERNDIMQA - ANALYTE IN DETAIL

Special assays serum 2021

Lab Name : **ERN0049 - HOSPITAL SAN JOAN DE DEU - Servei de Bioquimica (Metabolopaties)**

Method Set : HSJD

Sample : SAS2021.08

Analyte : Coenzyme Q10

Deadline : 29/10/2021

Unit : µmol/L

Your Method : HPLC-Electrochemical detection

Your Result : 0.510

Parameter	Method Results	All Labs Results
n :	5	13
Mean :	0.590	0.520
Median :	0.590	0.532
SD :	0.119	0.274

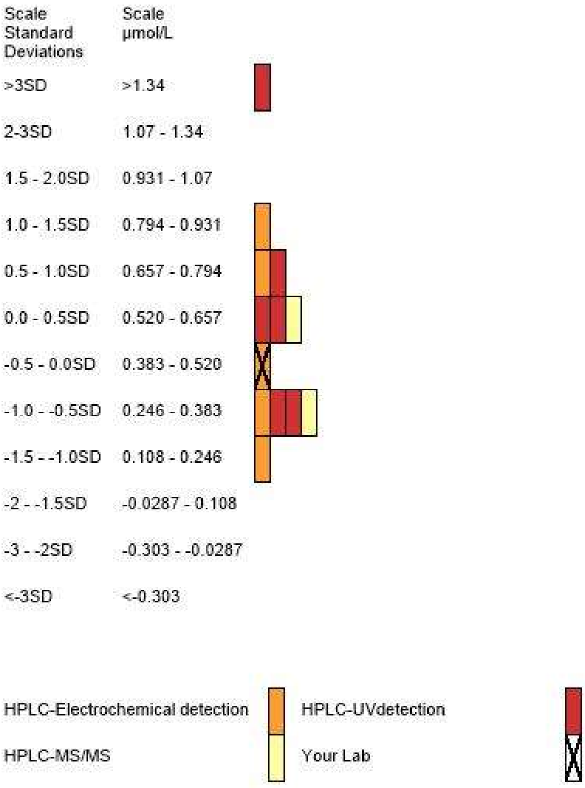

Supplement: Supplementary file 1 [file antioxidants-11-00528-s001.zip › Supplementary Material 2.pdf]
