# Supplementary material for: Technical Aspects of Coenzyme Q10 Analysis: Validation of a New HPLC-ED Method
Source: Antioxidants (Basel). 2022 Mar 10;11(3):528. doi: 10.3390/antiox11030528 (PMC8944485; doi:10.3390/antiox11030528)
Supplement: Supplementary file 1 [file antioxidants-11-00528-s001.zip › Supplementary Table S1.pdf]

Supplementary Table S1. Accuracy assessed by laboratory intercomparison.

Mean values detected (µmol/L)		
	**2020**	**2021**
All participants	0.757 (n = 11)	0.901 (n = 13)
New HPLC-ED method	0.860	1.34
**% deviation**	113.6	148.7
