# Supplementary figures and images for: Technical Aspects of Coenzyme Q10 Analysis: Validation of a New HPLC-ED Method
Source: Antioxidants (Basel). 2022 Mar 10;11(3):528. doi: 10.3390/antiox11030528 (PMC8944485; doi:10.3390/antiox11030528)

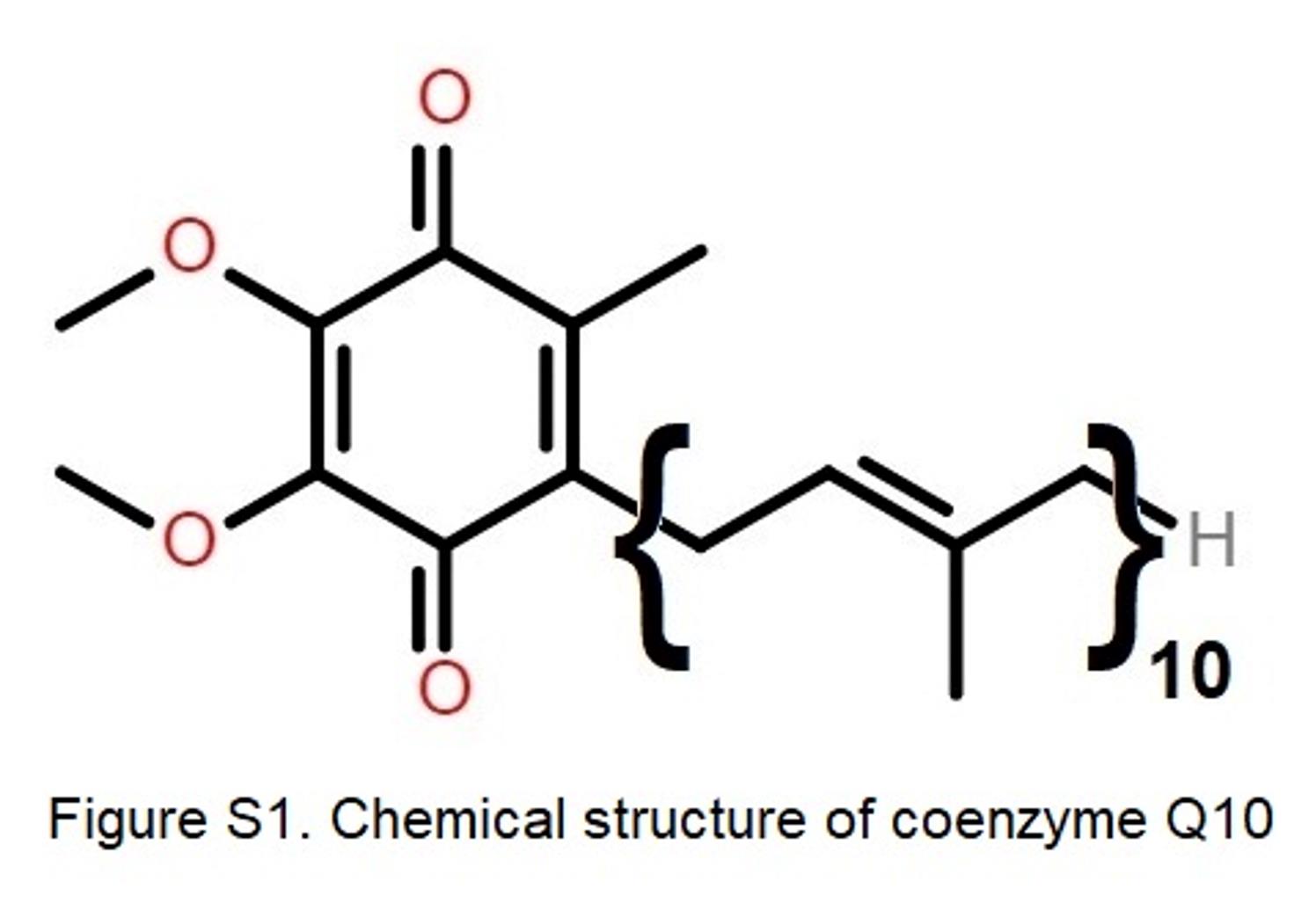

Supplement: Supplementary file 1 [file antioxidants-11-00528-s001.zip › Figure S1.jpg]

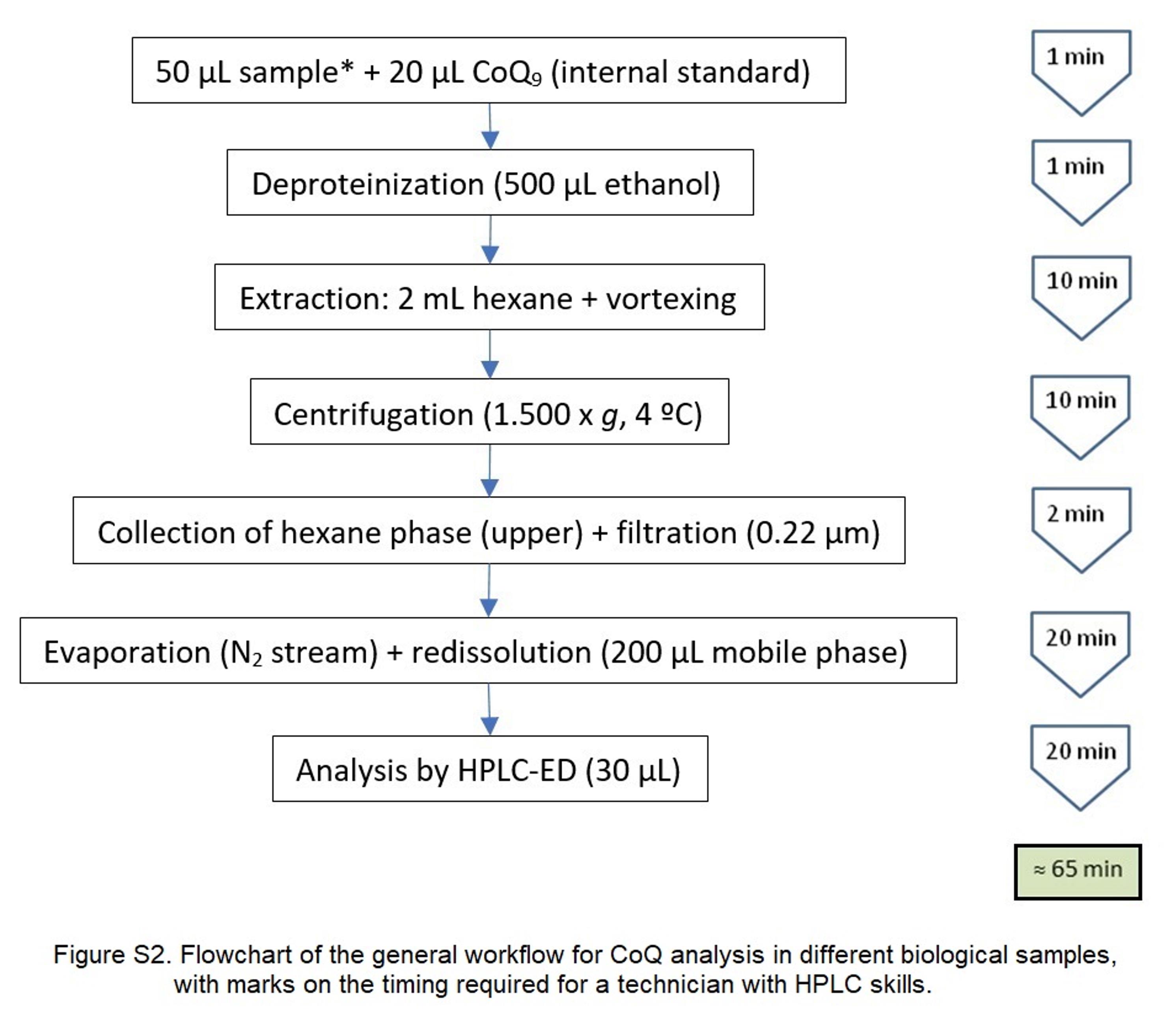

Supplement: Supplementary file 1 [file antioxidants-11-00528-s001.zip › Figure S2.jpg]

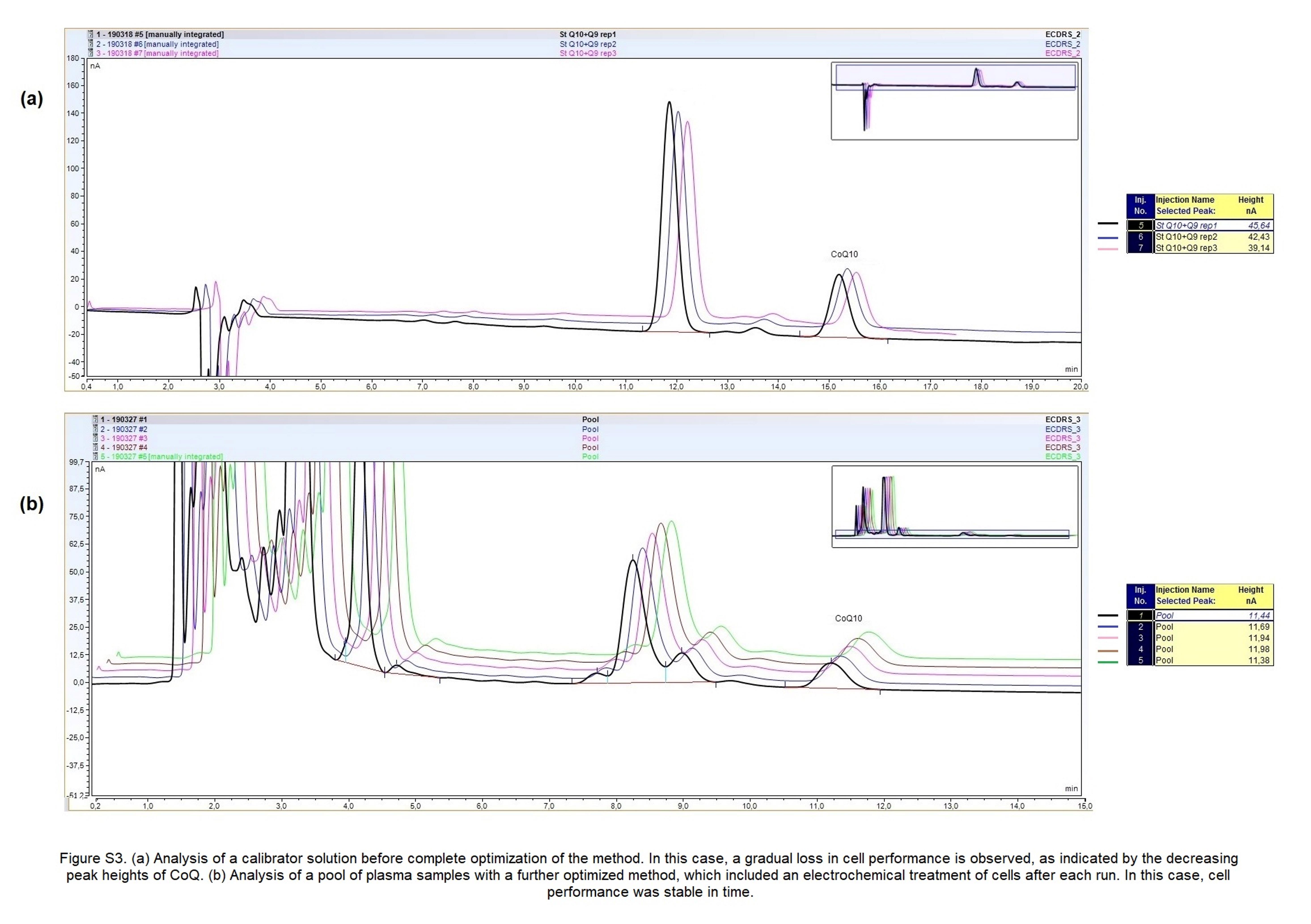

Supplement: Supplementary file 1 [file antioxidants-11-00528-s001.zip › Figure S3.jpg]

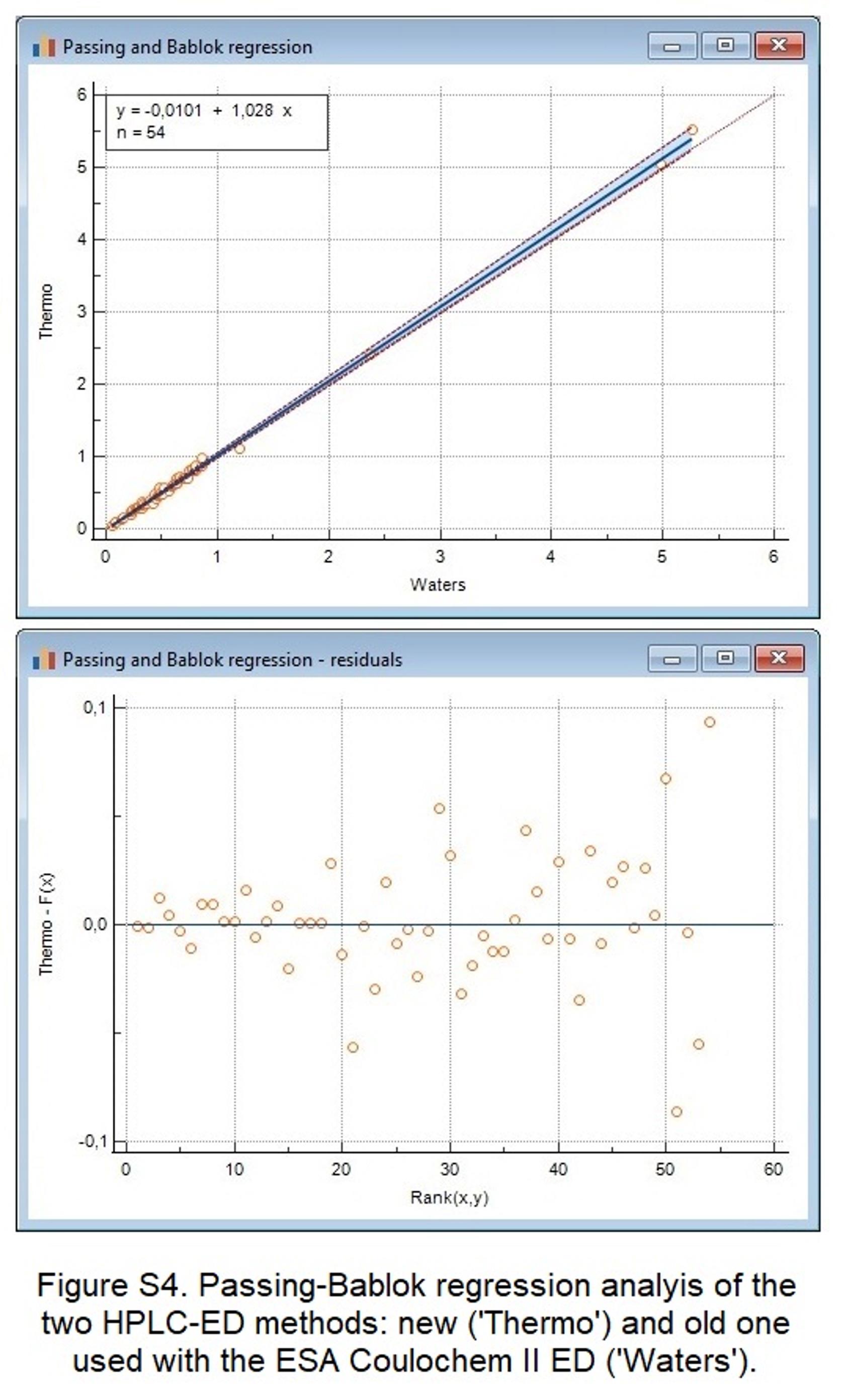

Supplement: Supplementary file 1 [file antioxidants-11-00528-s001.zip › Figure S4.jpg]
